# Adult-Onset Still's Disease with Dermatopathic Lymphadenitis Clinicopathologic Features: A Rare Case Report and Review of the Literature

**DOI:** 10.1155/2022/1653683

**Published:** 2022-06-03

**Authors:** Reda A. Elhawary, Mir Nadeem, Mohammed S. Abdelwahed, Mansour Somaily, Shahenda Y. Alemam

**Affiliations:** ^1^Department of Pathology, Faculty of Medicine, Al-Azhar University, Cairo, Egypt; ^2^Department of Pathology, Histopathology Consultant, Khamis Mushyte General Hospital, Khamis Mushait, Saudi Arabia; ^3^Department of Medicine, King Khalid University and Associated Hospital, Abha, Saudi Arabia; ^4^Department of Pathology, Faculty of Medicine, University of Jeddah, Jeddah, Saudi Arabia; ^5^Department of Medicine, Rheumatology Division, King Khalid University Medical City, Abha, Saudi Arabia; ^6^Department of Medicine, Rheumatology Division, Khamis Mushyte General Hospital, Khamis Mushait, Saudi Arabia

## Abstract

Adult-onset Still's disease (AOSD) is an inflammatory disorder characterized by fever, arthritis, and a transient skin rash. It is a rare condition characterized by inflammatory multisystem changes of unknown cause. A 35-year-old woman was admitted to rheumatology department of tertiary care hospital complaining of painful wrist and skin rash as well as fever, generalized lymphadenopathy, weight loss, and fatigue. The early diagnosis of AOSD was confirmed by clinical history, examination, and laboratory tests, as well as a confirmatory skin biopsy with typical histopathological features, namely, upper epidermal dyskeratosis and dermal inflammatory neutrophilic infiltration. The patient's condition was treated with steroids and NSAIDs, to which she responded well, and on follow-up, her symptoms regressed along with improvement in biochemical parameters. The authors suggest that skin biopsy and confirmation of histopathological diagnosis of AOSD are useful in the diagnosis and proper management of AOSD patients in cases with clinical suspicion of AOSD.

## 1. Introduction

Adult-onset Still's disease (AOSD) is a rare inflammatory disorder with a variety of clinical symptoms, including high-grade fever, arthritis, a transient rash, lymphadenopathy, and multiple organ involvement [1]. The annual incidence of AOSD has been estimated between 0.16 and 0.4 per 100,000 people worldwide, with the reported prevalence rates from 1 to 34 cases per 1 million people [2]. The incidence of AOSD remains low due to underdiagnosis of the condition and the insidious nature of the disease. Macrophage activation syndrome (MAS) and disseminated intravascular coagulation (DIC) are two life-threatening consequences of untreated AOSD [3]. Still's disease is named after the clinician George Still, who first described it in children in 1896. In 1971, another clinician, Bywater, documented a similar clinical presentation in 14 patients. Young adults are most commonly affected by the disease, which has a bimodal age distribution of 15–25 and 36–46 years [4]. Several classification criteria have been proposed; however, the Yamaguchi criteria [5] are the most widely cited and provide the highest sensitivity (93%) within these criteria, and the exclusion of other diseases is required. Fautrel et al. [6] proposed a new set of criteria that added serum ferritin and glycosylated ferritin levels, but no exclusion criteria were included. The sensitivity and specificity of Fautrel criteria are 80.6% and 98.5%, respectively. The outcome of AOSD is unpredictable, and the disease is conventionally divided into three different subtypes based on disease evolution: monophasic course, usually a single cyclic course, intermittent course, and, finally, chronic course. The condition in patients with monophasic course usually resolves within weeks to months and regresses completely in less than a year [7]. Glucocorticoids (GCs) commonly induce a dramatic clinical response, even within few hours and days; the higher dosages are reported as being more efficient than lower dosages leading to subsequent less relapses of the disease [8]. The reduction of concomitant glucocorticoids dosage following treatment with the IL-1 receptor antagonist is seen in adult-onset Still's disease [9]. Methotrexate and other disease-modifying antirheumatic drugs (DMARDs) are commonly used to treat severe acute inflammatory synovitis [10]. In addition to DMARDs, biologic agents are used to treat refractory AOSD, including TNF-*α* inhibitors (infliximab, etanercept) and IL-1 (anakinra, canakinumab) and IL-6 blockers (tocilizumab, sarilumab) [11].

## 2. Case Report

A 35-year-old woman presented to the rheumatology clinic of a tertiary care hospital in Aseer complaining of wrist pain and skin rash started 4 months ago. The patient had accompanying symptoms such as weight loss, fatigue, inability to climb stairs, and inability to raise her arm above her head. The patient had no significant past medical history. On examination, the patient had polyarthritis with bilateral arthritis of the hand (metacarpophalangeal, interphalangeal) and shoulder joints and a maculopapular erythematous rash affecting both extremities. After 2 days of admission, the patient developed a fever which was high grade, and the maximum recorded temperature of 102.4°F occurred in the late afternoon. Laboratory investigations revealed leukocytosis with TLC (16000/l), anemia (haemoglobin 9 gm/dl), elevated ESR (50 mm/h) and C-reactive protein (CRP 88 mg/l), and serum ferritin (8000 ng/ml), and CT scan of the thorax and abdomen and pelvis showed generalized lymphadenopathy without any significant infective focus with cervical lymphadenopathy largest one measuring 2.3*∗*3.2 cm in left submandibular region also bulky bilateral axillary lymph nodes largest measuring 3.3*∗*2.3 cm along with mediastinal and para-aortic lymph nodes. Rheumatoid factor (RF), anti-CCP, and antinuclear antibody (ANA) tests were negative. The differential diagnoses in addition to AOSD, at the preliminary evaluation, were mixed connective tissue disease, rheumatoid arthritis, and psoriatic arthritis.

**Figure 1:**
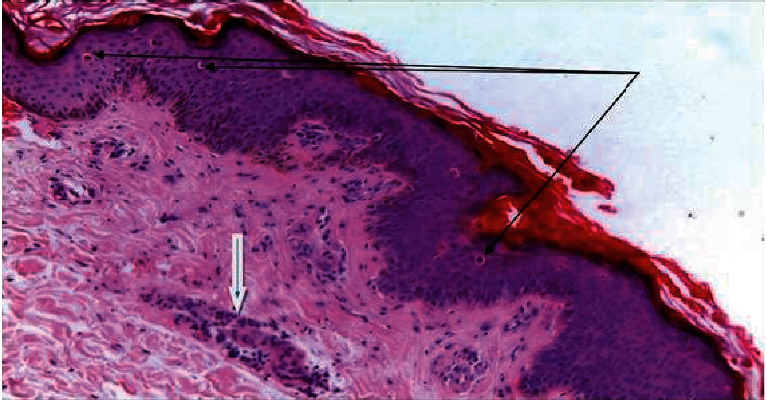
Adult onset Still disease, skin biopsy with multiple individual dyskeratotic keratinocytes in the upper epidermal layers (thin arrows) and perivascular neutrophilic inflammatory infiltrate (thick arrow) (H&E X100).

**Figure 2:**
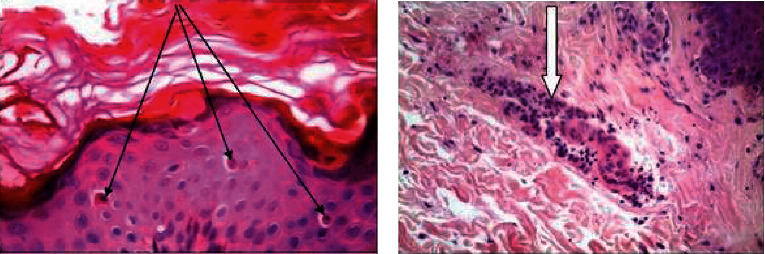
Adult onset Still disease, (A) skin biopsy with (a) multiple individual dyskeratotic keratinocytes in the upper epidermal layers (thin arrows) and (b) perivascular neutrophilic inflammatory infiltrate (thick arrow) (H&E X400).

**Figure 3:**
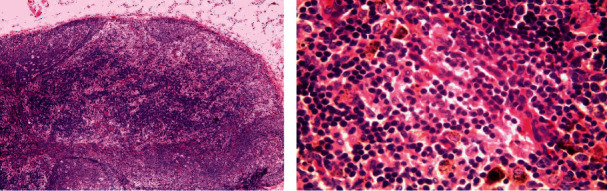
Dermatopathic lymphadenopathy. (a) Subcapsular nodular showing palely stained area in the paracortical region. Hyperplastic lymphoid follicles are also present (H&E X100). (b) Numerous pigment-containing histiocytes were present in the pale stained area (H&E X400).

Punch biopsy of the skin was done in coordination with the dermatology department in day care, and it revealed epidermal dyskeratotic keratinocytes mainly in the upper layers of the epidermis and focally in the stratum corneum (Figure 1). There was a superficial perivascular, periadnexal, and interstitial inflammatory infiltrate rich in neutrophils and few lymphocytes (Figure 2). These histologic features are consistent with Still's disease in adults. To rule out the possibility of lymphoma, a lymph node biopsy was performed, which revealed a preserved normal lymph node architecture, reactive lymphoid follicles, and marked paracortical expansion with irregular pale areas (Figure 3) consisting of numerous foamy histiocytes with brown melanin pigment, interdigitating dendritic cells, and Langerhans cells mixed with small lymphocytes.

Eventually, the patient was diagnosed with adult-onset Still's disease with associated dermatopathic lymphadenitis characterized as the monocyclic clinical pattern of AOSD. The patient was treated with anti-inflammatory drugs, a low dose of the steroid methylprednisolone 1 mg/kg for two weeks, and nonsteroidal anti-inflammatory drugs (NSAIDs) were administered because of the active arthritis. The patient improved after treatment, and upon two weekly follow-up for six months clinically, the patient had no fever, her arthritis improved, rash resolved, and ESR and C-reactive protein (CRP) were negative. Clinically, there was no lymphadenopathy with no further rheumatological complaints.

### 2.1. Ethical Declaration

The case report was conducted in full compliance with the Declaration of Helsinki and according to the research guidelines, and informed consent was obtained from the patient for publication as a rare case report. Ethical approval from the institution was not needed for case report publication.

## 3. Discussion

George Still in 1896 recognized a clinical picture in children that resembled polyarthritis in adults and named it Still's disease, which is the eponymous term for juvenile idiopathic arthritis. In 1971, a number of adult patients were observed to have features similar to children with juvenile arthritis and did not meet the criteria for classic rheumatoid arthritis, so the term adult Still's disease was coined to describe these patients [6]. The etiology of AOSD is unclear; however, genetic variables and various infectious triggers have been proposed as key etiologic features. However, confirmation of an infectious etiology is still under investigation, and the evidence for genetic components is inconclusive. There is uncertainty regarding the presence of the same etiopathogenic factors among all the AOSD patients, and it has always been a cause of concern. Various pathogens, including viruses and bacteria, have been studied to determine their involvement in the pathogenesis of AOSD. Bacterial pathogens include *Yersinia enterocolitica* and *Mycoplasma pneumonia* [8]. Studies have been conducted on the immunogenetics of AOSD, and a study on 62 French patients found an association between HLA-B17, -B18, -B35, and -DR2 and AOSD. However, the study has not been validated by a larger investigation [9]. Fever, rash, sore throat, and arthralgia are the common symptoms of AOSD [12]. Fever is usually above 39.0°C, and peak temperatures are observed in the late afternoon and early evening [13]. The normal rash in AOSD is asymptomatic and defined as salmon-colored, maculopapular eruptions affecting mainly the trunk and extremities. However, in active AOSD, an unusual, nonevanescent rash with pruritic, persistent papules or plaques has been observed in addition to the conventional rash [14]. Sore throat and an increase in serum ferritin are both important signs of AOSD and may be associated with odynophagia [15]. Several cytokines, including tumor necrosis factor-alpha (TNF-*α*), interleukin (IL)-6, and IL-18, have been associated with the pathophysiology of AOSD. The levels of these cytokines are extremely high in people with active AOSD [3]. The knees, wrists, ankles, and elbows are affected. During febrile episodes, joint symptoms usually worsen [16].

In our case, laboratory tests showed a high ESR (50) and leukocytosis (16000) with a preponderance of neutrophils. AOSD is characterized by a disproportionately high serum ferritin level (6000). Hyperferritinemia affects approximately 70% of patients and was previously considered to be due to cytokine production triggered by the reticuloendothelial system or liver injury [17]. As in our diagnostic case report, rheumatoid factor and antinuclear antibodies were usually negative [18]. AOSD as a diagnosis of exclusion required a thorough examination including skin biopsy, which was performed. Histopathologic examination of the rash in this case revealed dyskeratotic cells confined to the upper epidermis and stratum corneum, perivascular neutrophilic inflammatory infiltration of the upper dermis, and nuclear debris. The specific histological features of the rash in AOSD patients have been documented in several publications such as Cozzi et al. [19], with dyskeratosis confined to the upper epidermis and stratum corneum serving as an important diagnostic marker, as explained by neutrophil and lymphohistiocytic infiltration of the upper dermis and dermal mucin deposits are among the additional features. Parakeratosis, an interface dermatitis with basal vacuolization, or some necrotic keratinocytes are examples of epidermal changes. Although lymphadenopathy is a typical AOSD symptom, there are few studies on the histology of the lymph nodes, which are mostly case reports. The enlarged lymph nodes of this patient had reactive paracortical hyperplasia with dermatopathic changes on histological examination [20]. Other histopathological features include paracortical and diffuse hyperplasia and paracortical, follicular, and diffuse hyperplasia. Proliferation of arteries and lymph nodes and immunoblastic proliferation may be present. The pathologic differential diagnosis includes malignant lymphomas such as Hodgkin's lymphoma and angioimmunoblastic T-cell lymphoma (AITL). In AITL, the neoplastic T cells are medium to large in size. Large lymphoid cells with an abundant transparent cytoplasm, and Hodgkin's cells were not seen in our case [12, 21].

## 4. Conclusion

AOSD is a rare disease that requires a high clinical index of suspicion, and diagnosis of AOSD is difficult as there is no clinical or paraclinical ascertainment of diagnosis. We emphasize the important role of skin biopsy and histopathology in early diagnosis of this rare disease to achieve complete relief of symptoms and save the patient from severe complications.

## Data Availability

Data are available and can be obtained on request to the corresponding author.
